# Secondary batteries with multivalent ions for energy storage

**DOI:** 10.1038/srep14120

**Published:** 2015-09-14

**Authors:** Chengjun Xu, Yanyi Chen, Shan Shi, Jia Li, Feiyu Kang, Dangsheng Su

**Affiliations:** 1 Graduate School at Shenzhen, Tsinghua University, Shenzhen 518055, P. R. China; 2 State Key Laboratory of New Ceramics and Fine Processing, Department of Materials Science and Engineering, Tsinghua University, Beijing 100084, P. R. China; 3 Department of Inorganic Chemistry, Fritz Haber Institute of the Max Planck Society, Faradayweg 4-6, 14195 Berlin, Germany; 4 Shenyang National Laboratory of Materials Science, Institute of Metal Research, Chinese Academy of Sciences, Shenyang 110016, P. R. China

## Abstract

The use of electricity generated from clean and renewable sources, such as water, wind, or sunlight, requires efficiently distributed electrical energy storage by high-power and high-energy secondary batteries using abundant, low-cost materials in sustainable processes. American Science Policy Reports state that the next-generation “beyond-lithium” battery chemistry is one feasible solution for such goals. Here we discover new “multivalent ion” battery chemistry beyond lithium battery chemistry. Through theoretic calculation and experiment confirmation, stable thermodynamics and fast kinetics are presented during the storage of multivalent ions (Ni^2+^, Zn^2+^, Mg^2+^, Ca^2+^, Ba^2+^, or La^3+^ ions) in alpha type manganese dioxide. Apart from zinc ion battery, we further use multivalent Ni^2+^ ion to invent another rechargeable battery, named as nickel ion battery for the first time. The nickel ion battery generally uses an alpha type manganese dioxide cathode, an electrolyte containing Ni^2+^ ions, and Ni anode. The nickel ion battery delivers a high energy density (340 Wh kg^−1^, close to lithium ion batteries), fast charge ability (1 minute), and long cycle life (over 2200 times).

The use of electricity generated from clean and renewable sources, such as water, wind, or sunlight, requires efficient distributed electrical energy storage by high-power and high-energy secondary batteries using abundant, low-cost materials in sustainable processes^1^. The secondary batteries capable of storing enormous electric energy at a very large power are of importance for our society. Battery, whose chemistry is based on cathodic and anodic reactions occurring at the interface between the electrodes and electrolyte, generally composes of a cathode, an anode, an electrolyte and a separator^2^. Secondary battery is rare in battery industries because it is difficult to gather two electrochemically reversible cathodic and anodic reactions in one electrolyte as the battery chemistry. There are only several kinds of secondary (rechargeable) batteries in the world: lithium, lithium ion (LIB), sodium ion, nickel cadmium (Ni-Cd), lead-acid, magnesium, calcium and aluminum batteries^1^,^3^,^4^,^5^,^6^,^7^,^8^,^9^,^10^,^11^,^12^. Most of the current batteries, for example lithium ion batteries, utilize univalent ions (i.e. H^+^, Li^+^, Na^+^or K^+^) as media to store energy. Moreover, most of them are incapable of fast charge^8^,^13^. Here, we show “how to discover the secondary battery chemistry with the multivalent ions for energy storage” and report a new rechargeable nickel ion battery with fast charge rate. There are three steps for the fabrication of a battery. Firstly, we need choose two reversible reactions in one electrolyte as the cathodic and anodic reactions, respectively. Secondly, suitable cathode and anode materials are required to carry out these reactions. Finally the rechargeable battery is fabricated with certain cathode, anode, electrolyte, and a separator.

The multivalent ions, for example Mg^2+^ or Al^3+^ ion, are used for energy storage to fabricate magnesium or aluminum battery^10^,^11^,^12^,^14^,^15^,^16^,^17^. The investigation on the reversible intercalation of Mg^2+^ ions into Chevrel phase such as Mo_3_S_4_ indicated an extremely slow intercalation kinetics and low charge capacity^17^. It is generally believed that the kinetics of insertion of multivalent ions into host solid state electrode is much slower than that of univalent ions. While in MnO_2_-based supercapacitors the charge rate and capacitance of the host electrode material (for example MnO_2_) can be doubled by using multivalent Ca^2+^ ion compared with univalent Na^+^ ion^18^,^19^,^20^,^21^,^22^,^23^,^24^. By using trivalent La^3+^ ion, the charge rate and capacitance of MnO_2_ can be further improved^22^. The research in the supercapacitor application inspired us that we can set up suitable systems for multivalent ions to obtain high intercalation capacity and a fast charge rate. We absorbed the idea and use it in battery field to invent rechargeable batteries with high energy density and fast charge rate. We realized this idea by using the insertion of multivalent Zn^2+^ or Ni^2+^ ion into alpha type manganese dioxide to invent two rechargeable batteries with a very fast charge rate^23^. In this manuscript, we report the energetic nickel ion chemistry and nickel ion battery for the first time. The nickel ion battery generally uses an alpha type manganese dioxide cathode, an aqueous NiSO_4_ electrolyte, and Ni anode. The nickel ion battery displays a high energy density (340 Wh kg^−1^, close to that of lithium ion batteries), fast charge ability (1 minute) and long cycle life (over 2200 times).

## Results

The common view that the multivalent ion is unsuitable for energy storage at a fast rate is not correct. Below we show that the storage of multivalent ions in certain host material with a large tunnel structure is feasible both thermodynamically and kinetically. Table 1 shows that the ion dynamic diameters of the multivalent ions are close to that of the univalent ions. It enables the probability of multivalent ions to be stored in alpha type manganese dioxide (α-MnO_2_) with a tunnel diameter of 0.28 nm.

**Figure 1:**
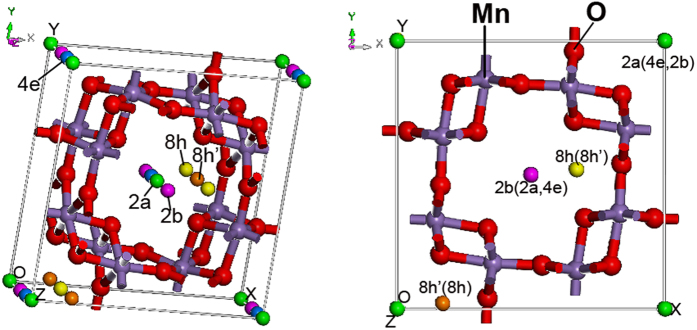
Possible positions (2a, 2b, 4e, 8 h’ and 8 h) in the tunnel of α-MnO_2_ for the insertion of univalent and multivalent ions. (The pictures are drawn by C. Xu and S. Shi).

We simulated the insertion of one ion in several possible positions (such as 2a, 2b, 4e, 8 h’ and 8 h) of one α-MnO_2_ tunnel by first principle calculation (Fig. 1)^9^,^25^. The summary of Li^+^, Na^+^, K^+^, Ni^2+^, Zn^2+^, Mg^2+^, Ca^2+^, Ba^2+^, or La^3+^ ion inserting in various positions can be seen in Table S1. The Li^+^, Na^+^, Zn^2+^, Mg^2+^, and Ca^2+^ ions favour inserting in the 8 h position of the tunnel, while Ni^2+^ ion favour the 2a position and K^+^, Ba^2+^ and La^3+^ ions favour the 2b position. The bond length of A–O ranges from 1.83 to 2.89 Å for different ion species (Table S1). When an ion inserts into the tunnel of α-MnO_2_, the tunnel slightly changes and the electrons are stored and shared by adjacent Mn and O atoms. The lowest binding energy (△*E*) after ion-insertion for each ion is listed in Table 1. The lower binding energy of multivalent “ion-insertion” indicates that insertion of multivalent ions is thermodynamically easier and more stable than insertion of univalent ions^9^,^25^.

Moreover, the kinetics of charge rate by using multivalent ion is faster than using univalent ion, which is directly seen as *nD*_0_ in Table 1. *D*_0_ represents the diffusion coefficient and n is the valence state of these cations. Most importantly, compared with univalent ion, the multivalent ions have the advantage that each multivalent ion inserting into α-MnO_2_ host results in the charge storage of over one electron (Figure S6). The lower required energy and faster charge rate of multivalent ions inserted into α-MnO_2_ enable the high capacity and fast charge ability for energy storage, which is also consistent with the experimental results in literature^18^,^20^,^21^,^22^,^23^.

**Figure 2:**
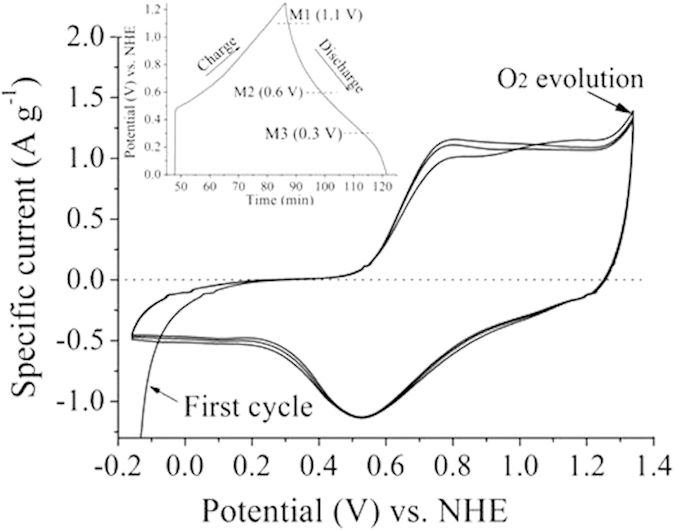
Cyclic voltammetric curve of α-MnO_2_ electrode at a sweep rate of 1 millivolt per second in 1.0 mol L^−1^ NiSO_4_ electrolyte. The insert shows the charge/discharge curve of α-MnO_2_ electrode in 1.0 mol L^−1^ NiSO_4_ electrolyte at a current density of 0.2 Ampere per gram.

In the first time, we experimentally found that each multivalent Ni^2+^, Zn^2+^, Mg^2+^, Ca^2+^, and Ba^2^ ion can electrochemically insert/extract into/from the tunnels of α-MnO_2_ as cathodic reaction.


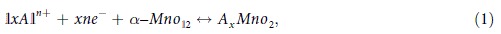


where A^*n*+^ represents Ni^2+^, Zn^2+^, Mg^2+^, Ca^2+^, Ba^2+^, or La^3+^ ion and *n* is the charge number^18^,^20^,^21^,^22^,^23^. The electrolyte refers to the aqueous solution of each multivalent ion with pH value ranging from 4.0 to 7.0, for example 1 mole per liter (mol L^−1^) Ba(NO_3_)_2_ or NiSO_4_ aqueous solution. Figure 2 shows the storage of each multivalent ion in α-MnO_2_. It can be seen that Ni^2+^ ion can insert/extract into/from α-MnO_2_ within a potential window ranging from 0.2 to 1.3 V (vs. NHE) reversibly. Insert of Fig. 2 shows the charge/discharge curves of reversible insertion/extraction of Ni^2+^ ion into/from α–MnO_2_. The results regarded to Zn^2+^, Mg^2+^, Ca^2+^, Ba^2+^, or La^3+^ ion have already been published and showed that the capacitance and charge rate of α-MnO_2_ could significantly double when replacing univalent Li^+^, Na^+^, or K^+^ ion with them^18^,^19^,^20^,^21^,^22^,^23^. In this paper, the fast reversible insertion/extraction of Ni^2+^ ion into/from α-MnO_2_ is firstly investigated. Most importantly, we use multivalent Ni^2+^ ion to invent a new rechargeable battery, named as nickel ion battery. Apart from α-MnO_2_, MnO_2_ samples with other typical tunnel structures, for instance, β-MnO_2_, γ-MnO_2_ and δ-MnO_2_, are also explored to store Ni^2+^ (See Part 1 in SI). However, a reversible intercalation process of Ni^2+^ ions only occurs in α-MnO_2_ due to its large and unique tunnel structure.

**Figure 3:**
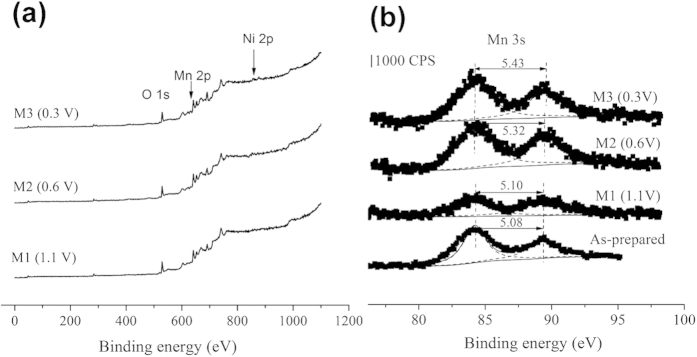
(**a**) The XPS survey of M1, M2 and M3 electrodes. (**b**) The Mn 3 s core level spectra of as-prepared, M1, M2 and M3 electrodes.

We further confirmed the storage of Ni^2+^ ions into α-MnO_2_ by X-ray photoelectron spectroscopy (XPS) and element mapping measurements. In order to explore the storage of Ni^2+^ ions into α-MnO_2_, individual α-MnO_2_ electrode is discharged from 1.25 to 1.1, 0.6, 0.3 V (denoted as M1, M2, and M3) by XPS and element mapping measurements, respectively. XPS analysis on the M1, M2, and M3 electrodes is employed to monitor the valence change of Mn and the molar ratio between nickel and manganese element after discharging (See part 2 in SI). The XPS survey of M1, M2 and M3 electrodes is shown in Fig. 3a. Figure 3b exhibits the Mn 3 s core levels for as-prepared, M1, M2, and M3 electrodes. The difference values of peak energies of the Mn 3 s are 5.08, 5.10, 5.32 and 5.43 eV for as-prepared, M1, M2, and M3 electrodes, respectively. These values are compared to 5.79, 5.50, 5.41, and 4.78 eV for reference samples of MnO, Mn_3_O_4_, Mn_2_O_3_, and MnO_2_, respectively^9^,^23^. A clear change of peak splitting of Mn 3 s was obtained during discharging, which indicates that the oxidation state of manganese changed from IV in oxidized state to III in reduced state. This plot shows that the average valence state of Mn decreased when the MnO_2_ electrode discharged, which means that a part of Mn(IV) ions is reduced to Mn(III) ion and the energy (electrons) is stored. Moreover, the oxidation state of alpha-MnO_2_ is almost the same after charge/discharge.

**Figure 4:**
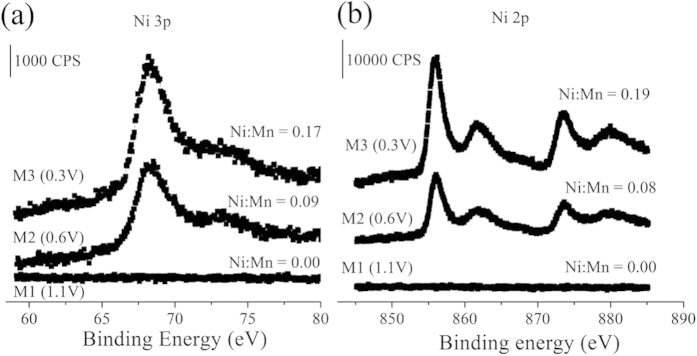
(**a**) Ni 3p and (**b**) Ni 2p core level spectra of M1, M2 and M3 electrodes.

**Figure 5:**
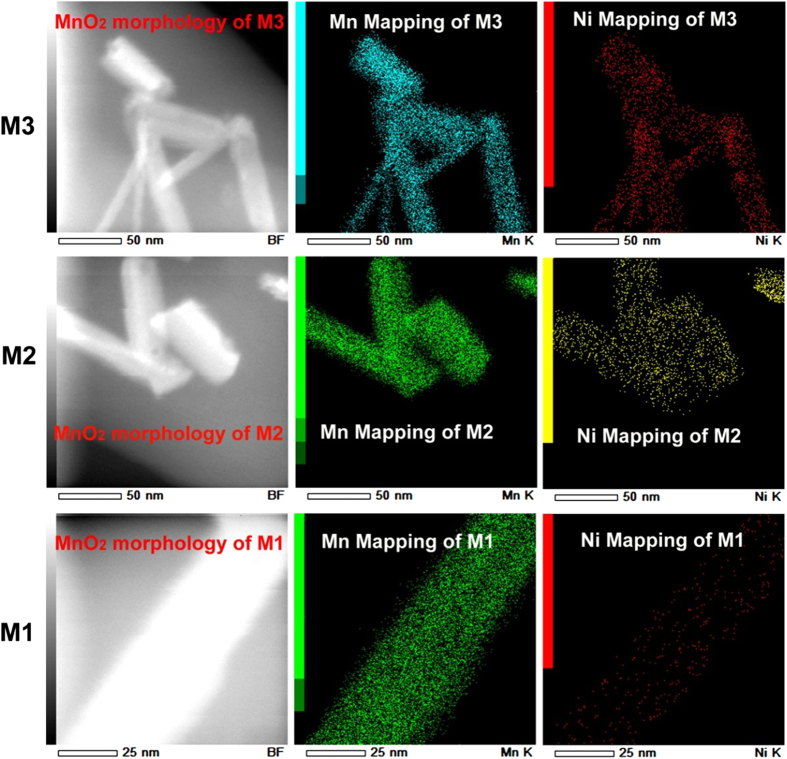
The morphology, Mn element mapping, and Ni element mapping of MnO_2_ in M1, M2, and M3 electrodes.

The Ni 3p and Ni 2p core level spectra of M1, M2 and M3 electrodes are shown in Fig. 4. As the potential decreases the molar ratio between Ni and Mn increases from 0.00 to 0.19, which clearly indicates the storage of Ni^2+^ ion in α-MnO_2_. The transmission electron microscope (TEM) mapping results of M1, M2, and M3 electrodes are shown in Fig. 5. It can be seen that the morphology of the α-MnO_2_ sample synthesized by the co-precipitation technique is rod-like shape with tens of nanometers in diameter. During discharging process, the electrode Ni ions are stored in MnO_2_. Therefore, results from XPS and TEM element mapping measurement directly demonstrated that Ni^2+^ ions are stored in α-MnO_2_. Furthermore, X-ray diffusion (XRD) measurement is used to monitor the structure change of MnO2 during the storage process of Ni^2+^ ions. It suggests that the main infrastructure of MnO_2_ has not been changed dramatically with the insertion of Ni^2+^ ions (Figure S7).

## Discussion

**Figure 6:**
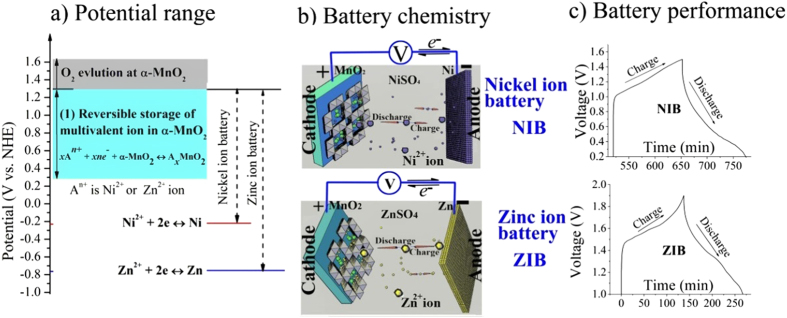
(**a**) The potential ranges of equations 2 to 3 (Solid lines). The dash lines show the maximum operating voltage of nickel ion battery (NIB) and zinc ion battery (ZIB). (**b**) The energetic chemistry of NIB and ZIB. NIB or ZIB use Ni^2+^ or Zn^2+^ ion as storage in both α-MnO_2_ cathode and Ni or Zn metal anode, respectively. (**c**) The charge/discharge curves of NIB and ZIB. This plot also shows the three steps to invent a rechargeable battery. (The pictures are drawn by C. Xu and S. Shi).

Generally, a battery composes of a cathode, an anode, an electrolyte and a separator. And battery chemistry is based on cathodic and anodic reactions occurring at the interface between electrode and electrolyte. We firstly discover the fast reversible insertion/extraction of Ni^2+^ ion into/from α-MnO_2_ in 1 mol L^−1^ NiSO_4_ aqueous solution as shown in equation 2. Therefore, the α-MnO_2_ electrode and 1 mol L^−1^ NiSO_4_ aqueous solution can be used as the cathode and the electrolyte, respectively. It also indicates that the reaction of equation 1 can be the cathodic reaction. Then reliable anode as well as anodic reaction has to be found for each multivalent ion to fabricate a full battery. Metal anode is the typical anode used in secondary battery chemistry. In the aqueous electrolyte, Ni metal is suitable for the anode. Deposition/dissolution of Ni^2+^/Ni occurs at ca. −0.23 V vs. NHE in 1 mol L^−1^ NiSO_4_ aqueous electrolyte (red line in Fig. 6a). The deposition of Ni^2+^ ion on Ni metal is found to be spherical shape with tens of nanometers in diameter (Figure S8).

With the α-MnO_2_ as cathode, Ni metal as anode, and 1 mol L^−1^ NiSO_4_ aqueous solution as electrolyte, we can invent a rechargeable battery. The battery chemistry is based on two electrochemically reversible cathodic and anodic reactions (equation 2 and equation 3). The potential ranges of cathodic reaction and anodic reaction are showed in Fig. 6a.


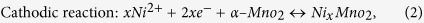



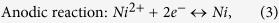


Subsequently, the energetic nickel ion chemistry as shown in Fig. 6b is proposed by using Ni^2+^ ion as the energy storage medium. Nickel ion battery composes of an α-MnO_2_ cathode, a nickel metal anode, and a NiSO_4_ mild aqueous electrolyte. During discharging process, anodic nickel is dissolved in the form of Ni^2+^ ion, which then inserts into α-MnO_2_ cathode, generating an electron current flow in the electrical loop and vice versa. Because the storage/release of energy is based on the migration of Ni^2+^ ion between cathode and anode, we name this battery as nickel ion battery (NIB).

In addition, Fig. 6 also shows the information of zinc ion battery (ZIB) to compare with NIB. ZIB is assembled by using α-MnO_2_ cathode, Zn anode and ZnSO_4_ aqueous electrolyte^23^. It can be seen from Fig. 6 that NIB and ZIB are similar in cathode due to the origin of the multivalent ion storage mechanism of α-MnO_2_. However, the electrolyte, anode and the most important battery chemistry are different.

**Figure 7:**
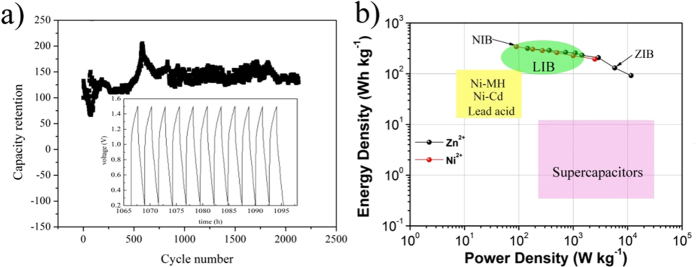
(**a**) Long cycle life of nickel ion battery at current density of 0.2 Ampere per gram. Inserts shows the charge/discharge curves of nickel ion battery. (**b**) Ragone plots of nickel ion battery (NIB) and zinc ion battery (ZIB) marked with line with dots, compared with state-of-the-art lead acid battery (Lead Acid), nickel cadmium (Ni-Cd), lithium ion battery (LIB) and supercapacitors marked with colorful area.

We assembled the prototype NIB (See Part 4 in SI) and ZIB (See Part 5 in SI), whose charge/discharge curves are shown in Fig. 6c. The NIB has a maximum operating voltage value of 1.5 V and delivers a capacity up to 298 milliampere hour per gram (mAh g^−1^) calculated based on the mass of MnO_2_. The long cycle life test has been performed on NIBs by the continuous galvanostatic charge/discharge at current densities of 200 milliampere per gram as shown in Fig. 7a. The continuous charge/discharge cycle curves of NIB are showed in the insert of Fig. 7a. After 2200 cycles NIB still shows a stable capacity and good columbic efficiency (Figure S13). The Ragone plots of NIB and ZIB are shown in Fig. 7b. It exhibits that these energy storage devices with multivalent Zn^2+^ or Ni^2+^ ions for energy storage cover a very wide range from batteries to supercapacitors and fill the gap between them.

Batteries with the multivalent ions for energy storage, for example NIB and ZIB, are capable of fast charge/discharge (1 minute) as shown in the insert of Fig. 7a and Figure S15. The energy densities of ZIB and NIB are estimated to be ca. 320 and 340 watt hours per kilogram (Wh kg^−1^) based on the weight of the total active mass of both cathode and anode. In the battery industry, the weight of the active mass of both cathode and anode is 30–50% of the total battery^13^. Therefore, the energy densities of total battery of ZIB and NIB should be up to 170 Wh Kg^−1^ and ca. 120 Wh Kg^−1^, which are higher than lead-acid, or Ni-Cd batteries (ca. 40 ∼ 80 Wh kg^−1^), and close to LIB (ca. 150 ∼ 400 Wh kg^−1^)^2^,^9^,^13^,^26^,^27^,^28^,^29^. In addition, NIB shows a longer cycle life over 2200 cycles than current aqueous batteries (ca. 1000 cycles)^2^,^30^. Furthermore, we have performed the nailing experiment on these batteries at a fully charged state. No sign of flash or smoke has been detected, which indicates the excellent safety. Therefore, these secondary batteries have great advantages in terms of safety, cycle life and energy density over the existing rechargeable batteries. These significant advantages enable the exploration of new industrial applications or to rival lead-acid or nickel cadmium batteries. For example, these secondary batteries are advanced candidates for hybrid electric vehicles or electric bicycles, or for storage of electricity generated from clean and renewable sources, such as water, wind or sunlight. This is just the beginning of searching for new batteries with multivalent ions. With the further exploration we anticipate that more rechargeable batteries with multivalent ions as energy storage will emerge.

In summary, we show that storage of multivalent Ni^2+^ or Zn^2+^ ion in alpha type manganese dioxide presents a stable thermodynamics and fast kinetics, which is confirmed by theoretic calculation and experiment confirmation. We further use storage process of the multivalent Ni^2+^ ions to discover a new battery chemistry and invent the rechargeable nickel ion battery. The secondary battery with multivalent Ni^2+^ ions for energy storage is advantageous in energy density (340 Wh kg^−1^), fast charge ability (1 minute), and long cycle life (over 2200 times). As multivalent ions are rich in quantity, we believe that the utilization of them may trigger a renaissance of new battery chemistry in the future.

## Methods

The MnO_2_ powder has been synthesized by a one step process or co-precipitation technique^19^ (See Methods in SI). Electrodes were prepared by mixing 70 wt% of MnO_2_ or MnO_2_/graphene as active material with 20 wt% of acetylene black (conductive additive) and 10 wt% of binder polytetrafluoroethylene (PTFE). 70 mg of MnO_2_ powder and 20 mg of acetylene black were firstly mixed and dispersed in ethanol by ultrasound for 30 min. Then the ink was dried at 80 °C for 4 h to get dark mixed powder and 10 mg of PTFE was added to get a paste with a few of ethanol. Then the paste was dried at 80 °C and a few of 1-methy-2-pyrrolidinone (NMP) were added to get a syrup. The syrup was cold rolled into thick films. Pieces of film with 1 ∼ 5 mg weight, typically 1 cm^2^ in size, were then hot-pressed at 80 °C under 100 MPa on a stainless steel mesh. The prototype nickel ion battery with α-MnO_2_ cathode, 1 mol L^−1^ NiSO_4_ aqueous electrolyte, a fiber paper separator and a nickel foam anode as shown in Figure S10. The nickel foam is used due to its larger surface area than plate.

Electrochemical tests were performed with Solartron 1480 electrochemical station and Land CT2001A equipment. The discharge capacity of the cell is calculated according to the formula:


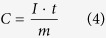


Where *C* is specific capacity (milliampere·hour per gram, mA·h g^−1^), *I* is the applied current (milliampere, mA), *t* is discharge time (hour, h), and *m* is the mass of the active material (gram). The energy density is calculated by the following equation:


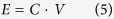


Where *C* is specific capacity (mA·h g^−1^) and V is the average voltage of battery. The average voltage of NIB and ZIB is 0.85 and 1.45 V.

The energy density of zinc ion battery (ZIB) and nickel ion battery (NIB) are listed in Table S2.

## Additional Information

**How to cite this article**: Xu, C. *et al.* Secondary batteries with multivalent ions for energy storage. *Sci. Rep.* **5**, 14120; doi: 10.1038/srep14120 (2015).

## Supplementary Material

Supplementary Information

## Figures and Tables

**Table 1:** Detailed information of univalent and multivalent ions.

Species	Diameter (Å)	*D*_*0*_ (×10^−14^m^−2^ s^−1^)	*nD*_*0*_ (×10^−14^m^−2^ s^−1^)	△*E*(eV)	Storage capacity (mAh g^−1^)
Li^+^	0.69	13.8	13.8	−5.006	63
Na^+^	1.02	11.6	11.6	−4.493	68
K^+^	1.38	7.3	7.3	−4.463	53
Mg^2+^	0.66	11.4	22.8	−5.601	97
Ca^2+^	0.99	9.8	19.6	−7.282	99
Ba^2+^	1.34	6.9	13.8	−7.308	81
Zn^2+^	0.74	4.3	8.6	−2.092	220
Ni^2+^	0.72	7.5	15.0	−5.54	298
La^3+^	1.06	3.7	11.1	−10.019	101
