# Supplementary material for: Secondary batteries with multivalent ions for energy storage
Source: Sci Rep. 2015 Sep 14;5:14120. doi: 10.1038/srep14120 (PMC4568479; doi:10.1038/srep14120)
Supplement: Supplementary Information [file srep14120-s1.doc]

**Electronic Supplementary Information**

**Secondary batteries with multivalent ions for energy storage**

*By Chengjun Xu, Yanyi Chen, Shan Shi, Jia Li, Feiyu Kang, Dangsheng Su*

**Methods**

1. **First principle simulation**

We stimulated the Li^+^, Na^+^, K^+^, Ni^2+^, Zn^2+^, Mg^2+^, Ca^2+^, Ba^2+^, or La^3+^ ion (denoted as A*^n^*^+^) insertion in α-MnO_2_ tunnels by CASTEP method in the possible positions of 2a, 2b, 4e, 8h' and 8h (See Figure 1). The summary of insertion of Li^+^, Na^+^, K^+^, Ni^2+^, Zn^2+^, Mg^2+^, Ca^2+^, Ba^2+^, or La^3+^ ion in various positions can be seen Table S1. Table 1 shows the binding energy and storage capacity of the insertion of Li^+^, Na^+^, K^+^, Ni^2+^, Zn^2+^, Mg^2+^, Ca^2+^, Ba^2+^, or La^3+^ ion in MnO_2_. In addition, the diameter, and diffusion coefficient are exhibited.

The binding energy (△E) is calculated by the following equation:

△E = E_AMn4O8_-E_Mn4O8_-E_A_ (S-1)

Where E_AMn4O8_ is the energy after insertion of ion A into Mn_4_O_8_, E_Mn4O8_ is the energy of Mn_4_O_8_, and E_A_ is the energy of the ion A.

Table S1 Summary of ion insertion into α-MnO_2_

|  | Position | O-A Bond length（Å） | E_AMn4O8_ (eV) | V_AMn4O8_ ^a^  (Å^3^) | E_A_  (eV) | △E  (eV) | △V^b^  (Å^3^) |
| --- | --- | --- | --- | --- | --- | --- | --- |
| Mn_4_O_8_ |  |  | -6105.075 | 143.526 |  |  | -- |
| Li | 2a | 2.32 | -6297.815 | 135.735 | -188.21 | -4.53 | -7.791 |
|  | 2b | 2.77 | -6297.531 | 136.691 | -188.21 | -4.246 | -6.835 |
|  | 4e | 2.52 | -6297.690 | -- | -188.21 | -4.405 |  |
|  | 8h' | 1.907, 1.926 | -6298.248 | 144.879 | -188.21 | -4.963 | 1.353 |
|  | 8h | 2.205 | -6298.291 | 141.274 | -188.21 | -5.006 | -2.252 |
| Na | 2a | 2.47 | -7412.54 | 140.0579 | -1303.2 | -4.265 | -3.468 |
|  | 2b | 2.84 | -7412.664 | 140.048 | -1303.2 | -4.389 | -3.478 |
|  | 4e | 2.63 | -7412.600 | -- | -1303.2 | -4.325 | -- |
|  | 8h' | 2.41, 2.44, 2.47, 2.69 | -7412.748 | 141.558 | -1303.2 | -4.473 | -1.968 |
|  | 8h | 2.69 | -7412.768 | 142.917 | -1303.2 | -4.493 | -0.609 |
| K | 2a | 2.6 | -6887.460 | 145.595 | -778.39 | -3.995 | 2.069 |
|  | 2b | 2.89 | -6887.767 | 142.706 | -778.39 | -4.302 | -0.82 |
|  | 4e | 2.9 | -6887.856 | -- | -778.39 | -4.391 | -- |
|  | 8h' | 2.595, 2.6×2, 2.604 | -6887.461 | 145.559 | -778.39 | -3.996 | 2.033 |
|  | 8h | -- | -6887.928 | 143.854 | -778.39 | -4.463 | 0.328 |
| Mg | 2a | 2.07 | -7082.580 | 130.275 | -972.59 | -4.915 | -13.252 |
|  | 2b | 2.41 | -7082.249 | 122.601 | -972.59 | -4.584 | -20.926 |
|  | 4e | 2.47 | -7081.39 | -- | -972.59 | -3.725 | -- |
|  | 8h' | 1.93, 1.96 | -7082.409 | 149.024 | -972.59 | -4.744 | 5.498 |
|  | 8h | 2.06 | -7083.266 | 146.751 | -972.59 | -5.601 | 3.225 |
| Ca | 2a | 2.31 | -7111.365 | 139.745 | -999.65 | -6.64 | -3.781 |
|  | 2b | 2.532 | -7111.958 | 128.790 | -999.65 | -7.233 | -14.736 |
|  | 4e | 2.63 | -7110.937 | -- | -999.65 | -6.212 | -- |
|  | 8h' | 2.238, 2.272, 2.509, 2.897 | -7111.169 | 137.683 | -999.65 | -6.444 | -5.843 |
|  | 8h | 2.623 | -7112.007 | 133.912 | -999.65 | -7.282 | -9.614 |
| Ba | 2a | 2.546 | -6808.585 | 149.213 | -697.55 | -5.96 | 5.687 |
|  | 2b | 2.81 | -6809.841 | 142.565 | -697.55 | -7.21635 | -0.962 |
|  | 4e | 2.845, 2.85 | -6809.380 | -- | -697.55 | -6.755 | -- |
|  | 8h' | 2.62×2,  2.653, 2.656 | -6809.059 | 143.862 | -697.55 | -6.434 | 0.336 |
|  | 8h | -- | -6809.933 | 142.998 | -697.55 | -7.308 | -0.528 |
| Sr | 2a | 2.436 | -6946.040 | 145.021 |  |  | 1.495 |
|  | 2b | 2.678 | -6946.9695 | 136.307 |  |  | -7.219 |
|  | 4e | 2.798, 2.81 | -6946.140 |  |  |  | -- |
|  | 8h' | 2.54, 2.55, 2.577, 2.62 | -6946.366 | 138.850 |  |  | -4.676 |
|  | 8h | -- | -6946.849 | 135.072 |  |  | -8.454 |
| Zn | 2a | 2.07 | -7816.190 | 128.985 | -1709.18 | -1.935 | -14.541 |
|  | 2b | 2.88 | -7815.310 | 139.940 | -1709.18 | -1.055 | -3.586 |
|  | 4e | 2.51 | -7815.780 | -- | -1709.18 | -1.525 | -- |
|  | 8h' | 1.869, 1.871 | -7816.154 | 147.432 | -1709.18 | -1.899 | 3.906 |
|  | 8h | 2.14 | -7816.347 | 145.602 | -1709.18 | -2.092 | 2.076 |
| Ni | 2a | 1.95 | -7460.325 | 123.940 | -1349.71 | -5.54 | -19.586 |
|  | 2b | 2.714 | -7457.708 | 133.407 | -1349.71 | -2.923 | -10.119 |
|  | 4e | 2.47 | -7458.510 | -- | -1349.71 | -3.725 | -- |
|  | 8h' | 1.843, 1.848 | -7459.366 | 146.782 | -1349.71 | -4.581 | 3.256 |
|  | 8h | 2.22 | -7459.117 | -- | -1349.71 | -4.332 | -- |
| La | 2a | 2.417 | -6971.980 | 146.453 | -858.44 | -8.465 | 2.926 |
|  | 2b | 2.607 | -6973.534 | 134.702 | -858.44 | -10.019 | -8.824 |
|  | 4e | 2.734, 2.745 | -6972.380 | -- | -858.44 | -8.865 | 0 |
|  | 8h' | 2.51, 2.529, 2.537, 2.577 | -6972.529 | 140.008 | -858.44 | -9.014 | -3.518 |
|  | 8h | -- | -6973.506 | 134.844 | -858.44 | -9.991 | -8.682 |
| *^a^* Volume of Mn_4_O_8_ after insertion of one ion  *^b^* Volume change of Mn_4_O_8_ after and before insertion of one ion | | | | | | | |

1. **Preparation of α-MnO_2_**

The MnO_2_ powder has been synthesized by a one step process or co-precipitation technique. Detailed information about synthesis process or physicochemical characterizations of MnO_2_ as well as preparation and electrochemical measurements of single electrodes could be seen in reference (*J. Electrochem. Soc.2009, 156(1): A73*). One step process is shown below. A 0.1 mol L^-1^ KMnO_4_ aqueous solution was prepared by dissolving potassium permanganate (AR, 99%) in deionized water. Meanwhile 13.32 g surfactant of high purity sodium bis(2-ethylhexyl) sulfosuccinate (Aerosol-OT, AOT) was added in 300 mL isooctane (oil) and stirred well to get an optically transparent AOT/isooctane solution. Then 16.2 mL of 0.1 mol L^-1^ KMnO_4_ solution was added in AOT/isooctane solution, and this solution was dispersed by ultrasound for 30 min to prepare a dark brown precipitate. The product was separated, washed copiously several times with distilled water and ethanol, and dried at 80 ℃ for 12 h. In order to improve the conductivity of the MnO_2_, we synthesized MnO_2_/graphene composite. 13.32 g AOT and 10 mg graphene was added in 300 mL isooctane (oil) and stirred well to get an well-dispersed mixture solution. Then 16.2 mL of 0.1 mol L^-1^ KMnO_4_ solution was added in mixture solution, and this solution was dispersed by ultrasound for 30 min to prepare a dark brown precipitate. The MnO_2_/graphene composite delivers the highest capacity up to 298 mAh g^-1^.

Co-precipitation technique is shown below. A 0.1 mol L^-1^ KMnO_4_ solution was prepared by dissolving potassium permanganate (AR, 99%) in deionized water, while stirring the solution, a 0.15 mol L^-1^ Mn(CH_3_COO)_2_ was quickly added. The KMnO_4_/ Mn(CH_3_COO)_2_ molar ratio was 2:3. A dark brown precipitate was immediately obtained according to:

2Mn(Ⅶ) + 3Mn(Ⅱ) → 5Mn(Ⅳ) (S-2)

The solution was then stirred for 4 h until the reaction completed. The product was separated, washed copiously several times with double distilled water, and dried at 80 ℃ for 12 h.

Above MnO_2_ samples were heat-treated at 300 °C in air for 10 h to synthesize crystalline α-MnO_2_.

1. **Characterization of single MnO_2_ electrode**

Electrochemical tests were performed with an Im6e (Zahner) electrochemical station. Three electrodes assembly is employed to perform the electrochemical measurements. The MnO_2_ electrodes, activated carbon electrodes, Zn plate, or Ni plates are assembled as the working electrode. A piece of platinum gauze and Hg/Hg_2_SO_4_ (in saturated K_2_SO_4_) were assembled as the counter and reference electrode. The potential is expressed by standard hydrogen electrode (NHE). The cyclic voltammetry was carried out at sweep rates ranging from 1 to 50 mV s^-1^. Galvanostatic charge-discharge cycling experiments were performed at current densities ranging from 0.1 to 1 A g^-1^.

For X-ray photoelectron spectroscopy (XPS), transmission electron microscopy (TEM) or X-ray diffraction (XRD) analysis, MnO_2_ electrodes were firstly charged to 1.25 V versus NHE in 1 mol L^-1^ Ni(NO_3_)_2_ electrolyte and then discharged to 1.1, 0.6, 0.3 V as shown in Figure 2, respectively. The MnO_2_ electrodes being discharged at1.1, 0.6, and 0.3 V (denoted as M1, M2, and M3). After discharging, the electrode films were firstly detached, washed copiously several times with distilled water, and immerged in distilled water for 12 h. Then the films were washed copiously several times with distilled water again and dried at 80 ℃. These films subsequently were subjected to XPS, TEM and XRD test.

**Part 1 Influence of tunnels of MnO_2_**

The structural frameworks of MnO_2_ consist of MnO_6_ octahedral subunits sharing vertices and edges to form endless chains, which can in turn be linked to neighboring octahedral chains by sharing corners or edges. The piling up of MnO_6_ units enables the building of one dimensional (1D), two dimensional (2D) or three dimensional (3D) tunnels. The different structures could be described by the size of their tunnel determined by the number of octahedral subunits T(*n*×*m*), as shown in Figure S1. Typically, and in nature, these tunnels of manganese dioxides can be filled with foreign cations, usually univalent or bivalent cations, or water molecular. The tunnel of MnO_2_ has long been used to store univalent cations (H^+^, Li^+^, Na^+^, or K^+^) for energy storage applications, such as Zn/MnO_2_ batteries, lithium ion battery, supercapacitor, etc. *γ*-MnO_2_ with T_1,2_ tunnels is a famous electrode material to store protons or lithium ions for Zn/MnO_2_ batteries.


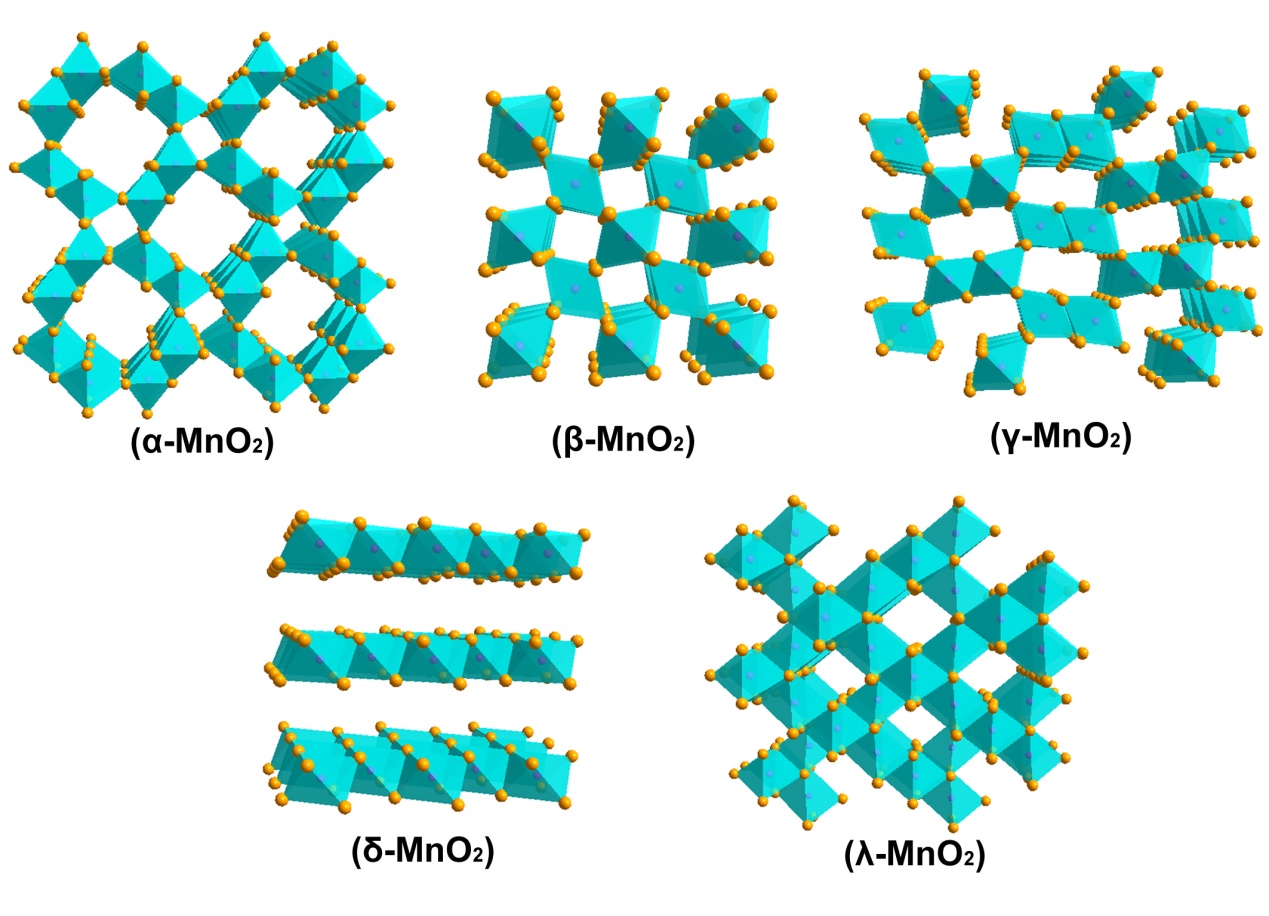


**Figure S1** Crystallographic structures of α- MnO_2_, β-MnO_2_, γ-MnO_2_, δ-MnO_2_, and λ-MnO_2_ (This picture is drawn by C. Xu and S.Shi)

We have synthesized MnO_2_ samples with typical tunnel structures, for instants, β-MnO_2_, α-MnO_2_, γ-MnO_2_, and δ-MnO_2_. We have also investigated the electrochemical behavior of MnO_2_ with different tunnels in 1 mol L^-1^ NiSO_4_ and found that the reversible intercalation process of Ni^2+^ ions only occurs in α-MnO_2_.

**1. α-MnO_2_**

Among all of MnO_2_, α-MnO_2_ possesses following merits:

1. It possesses a large open tunnel (2×2) structure;
2. It can contain very big cations, for example Ba^2+^, K^+^, Pb^2+^, etc,
3. Its tunnel is very stable with or without cations (water molecular can hold the tunnel structure);

The tunnel structure of α-MnO_2_ is shown in Figure S1. We have firstly synthesized the so-called standard amorphous α-MnO_2_ (See methods), which is a famous electrode material for supercapacitor applications. After heat-treatment at 300 ℃ for 12 h, crystalline α-MnO_2_ has also obtained. XRD pattern of amorphous and crystalline α-MnO_2_ is shown in Figure S2.


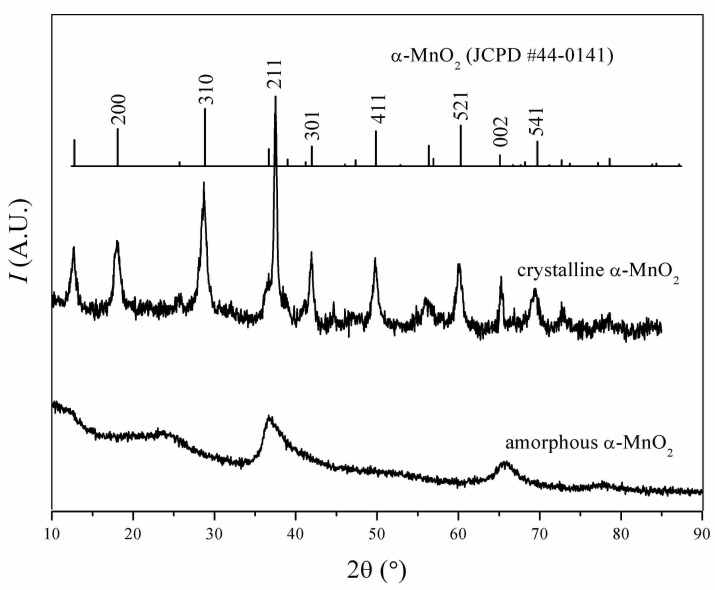


**Figure S2** XRD pattern of amorphous and crystalline α-MnO_2_

The cyclic voltammogram (CV) plot of α-MnO_2_ is shown in Figure 2, indicating that Ni^2+^ ions can reversibly intercalate into α-MnO_2_.

**2. Other MnO_2_**

We have investigated the effect of the tunnel structure of MnO_2_, including α-MnO_2_, β-MnO_2_, γ-MnO_2_, and δ-MnO_2_ in Ni(NO_3_)_2_ electrolyte.

β-MnO_2_ possesses a small tunnel (1×1). XRD pattern of β-MnO_2_ is shown in Figure S3a. Figure S3b shows CV plot of β-MnO_2_ at 0.5 mV s^-1^, which is comparable with that of α-MnO_2_. It is shown that intercalation process of Ni^2+^ ions does not occur, which should attribute to the small tunnel of β-MnO_2_.


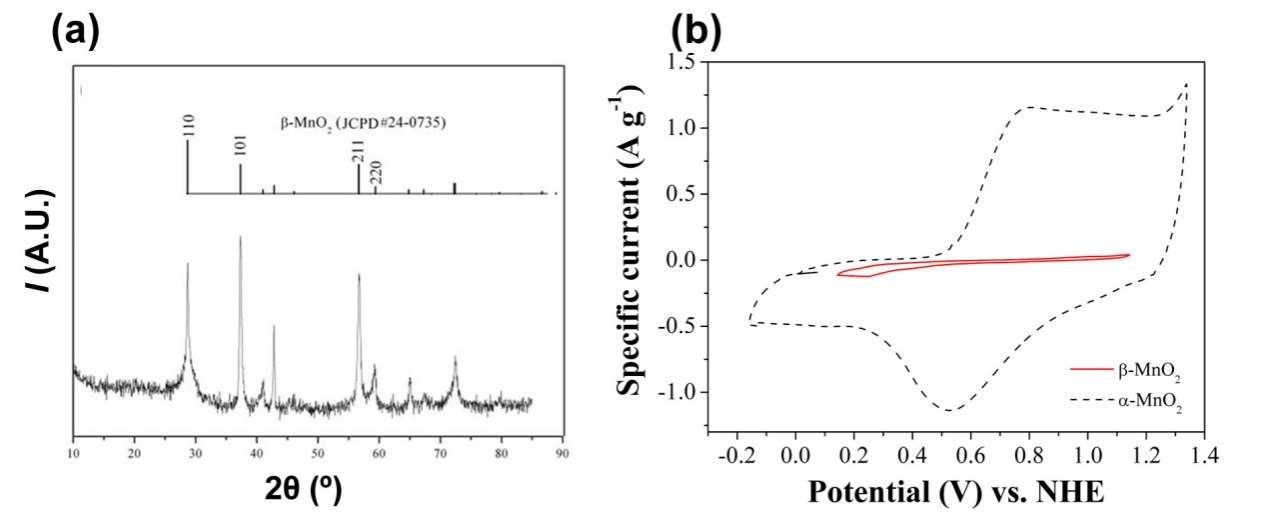


**Figure S3** (a) XRD pattern and (b) CV plot of β-MnO_2_

γ-MnO_2_ possesses a mix tunnel of (1×1) and (1×2). XRD pattern of γ-MnO_2_ is shown in Figure S4a. Figure S4b shows CV plot of γ-MnO_2_ at 0.5 mV s^-1^, which is compared with that of α-MnO_2_.


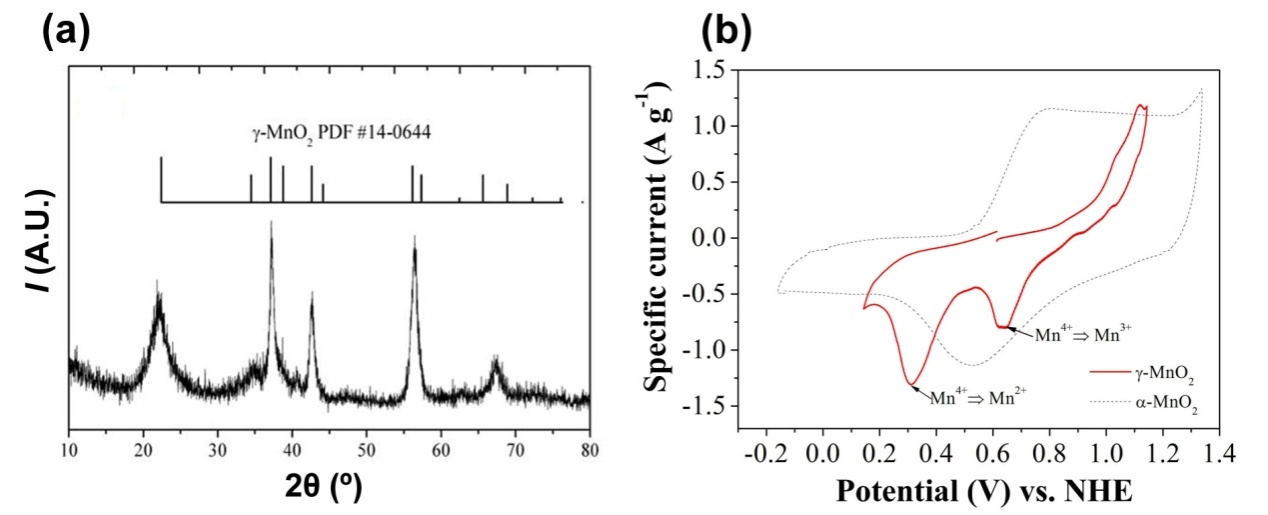


**Figure S4** (a) XRD pattern and (b) CV plot of γ-MnO_2_

δ-MnO_2_ possesses a layer structure with (1×∞) tunnel. XRD pattern of δ-MnO_2_ is shown in Figure S5a. Figure S5b shows CV plot of δ-MnO_2_ at 0.5 mV s^-1^, which is compared with that of α-MnO_2_.


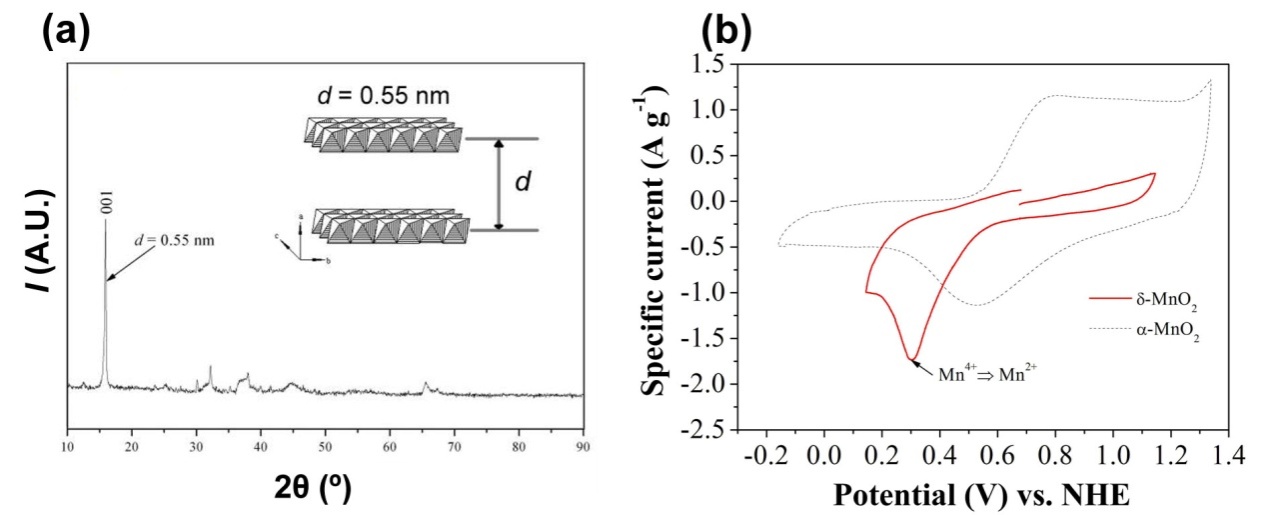


**Figure S5** (a) XRD pattern and (b) CV plot of δ-MnO_2_

CV plots of γ-MnO_2_ and δ-MnO_2_ are different from that of α-MnO_2_, which indicates a different mechanism. The electrochemical mechanism of γ-MnO_2_ and δ-MnO_2_ in aqueous solutions has been formerly proved to be two-electron pathways, i.e,

First electron process: (γ- or δ-)MnO_2_ + H^+^ + e^-^ → MnOOH (S-3)

Second electron process: MnOOH + H^+^ + e^-^ → Mn^2+^ +2H_2_O (S-4)

In summary, the γ-MnO_2_ and δ-MnO_2_, which is widely used in Zn/MnO_2_ battery, are both active to proton rather than Ni^2+^ cation and still follow the so-called two-step pathways in a mild electrolyte. For β-MnO_2_ and λ-MnO_2_, their small tunnels hinder the diffusion of Zn^2+^ ions. It is shown that a reversible intercalation process of Ni^2+^ ions only occurs in α-MnO_2_ due to its large and unique tunnel structure.

**Part 2 Storage mechanism of the multivalent cations stored in positive α-MnO_2_ in the mild electrolytes**

**Storage mechanism**

As shown in Figure S6, if one Ni^2+^ cation inserted into the MnO_2_ matrix, consequently two Mn^4+^ ions will be reduced to Mn^3+^ ion and two electrons will be stored.


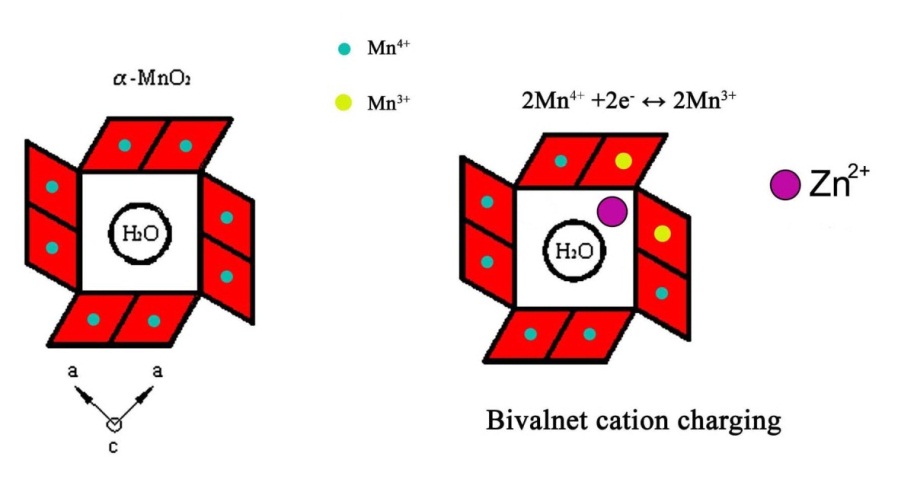


Ni^2+^

**Figure S6** Schematic graph of Ni^2+^ ion intercalation process in α-MnO_2_ with (2×2) tunnel.

**XPS analysis**

The Mn 3s core level spectra should usually show a peak splitting and a doublet due to the parallel spin coupling of 3s electron with the 3d electron during the photoelectron ejection. Such an exchange between electrons in the 3s-3d level of manganese occurs as a representative multiplet splitting and the corresponding separation of peak energies (△*E*) is described in terms of exchange interaction energy as:

△*E* = (2*S* + 1) *K*[3s, 3d], (S-5)

where S is the total spin of unpaired electrons in the 3s and 3d levels in the final states and *K*[3s, 3d] is the exchange integral between of 3s-3d energy levels. Consequently, the lower valence of Mn gives wider splitting of Mn 3s peaks.

The Mn 3s of as-prepared sample is showed in Figure 7a. The separation of peak energies of the Mn 3s peaks is about 5.07 eV for as-prepared MnO_2_ electrode.


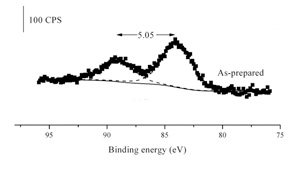

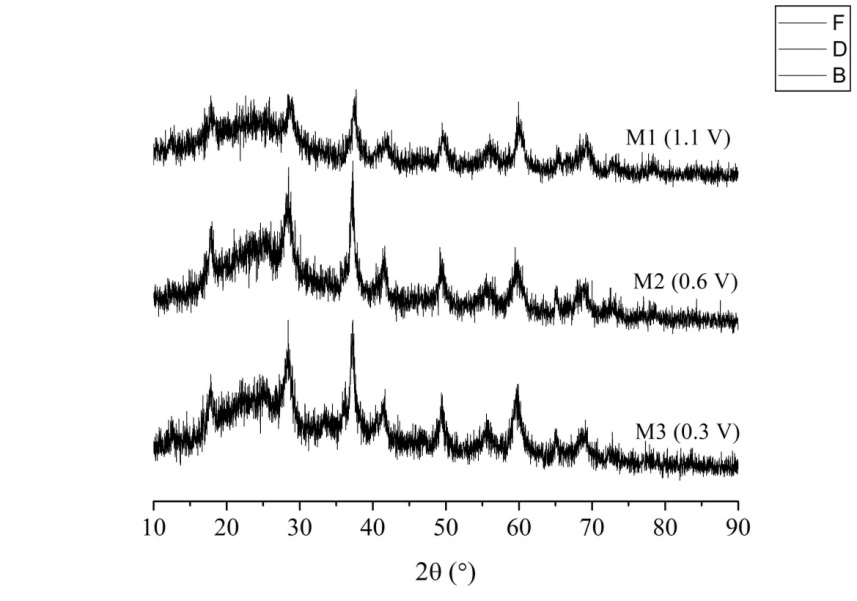


**(a)**

**(b)**

**Figure S7** (a) Mn 3s of as-prepared MnO_2_, (b) XRD patterns of M1, M2, and M3 electrodes.

**XRD analysis**

The MnO_2_ electrodes of M1, M2, and M3 have been suffered from X-ray diffusion (XRD) measurement to monitor the structure change of MnO_2_ during the storage process of Ni^2+^ ions. The XRD patterns of M1, M2, and M3 electrodes are shown in Figure S7 (b). As the decrease of the potential there is no significant difference between the patterns of M1, M2, and M3 electrodes, which shows the α-MnO_2_ profile (PDF No. 44-0141). It means that with the insertion of Ni^2+^ ions the main infrastructure of MnO_2_ has not been changed dramatically, which may be the reason of the long cycle life of nickel ion battery.

Therefore, it has been proved directly by XPS and element mapping that Ni^2+^ ions are stored in α-MnO_2_ as expressed as:


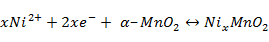
 (S-6)

**Part 3 Performance of negative nickel in the mild electrolytes**

In a mild aqueous solution containing Ni^2+^ ions, the dissolution/deposition process of nickel can be described as following equation:

Ni ↔ Ni^2+^ +2e^-^ (S-7)

The morphologies of nickel plates being charged/discharged for various cycles have shown in Figure S8. It is shown that after cycles the deposition of Ni occurs on the plate. The deposition of Ni^2+^ ion on Ni metal in 1 mol/L NiSO_4_ aqueous electrolyte is found to be spherical shape with tens of nanometers in diameter. After cycles, a loose layer has been deposited on the original smooth surface of nickel anode, which indicated that the deposition and dissolution of nickel occurs during charge and discharge.


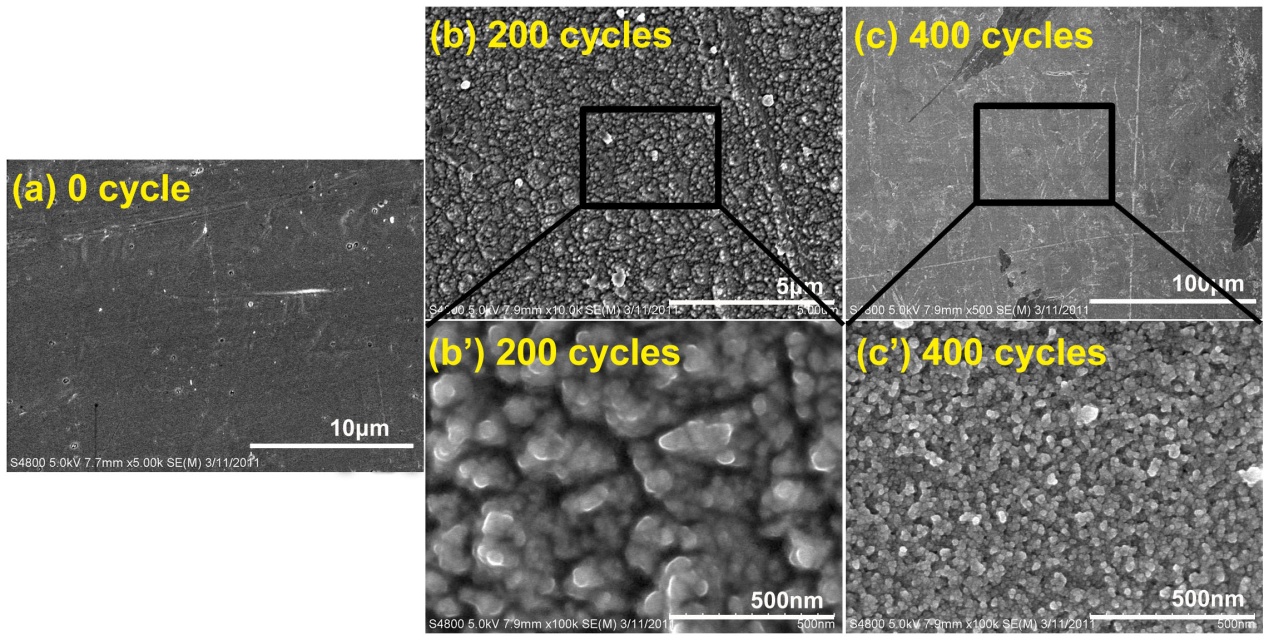


**Figure S8** Morphology of nickel plate being cycled for a) 0, b) 200, and c) 400 cycles

The XRD patterns of nickel anode before and after cycles are shown in Figure S9. It shows that the XRD patterns are identically similar for two samples. The XRD pattern is accordance to pure nickel.

**
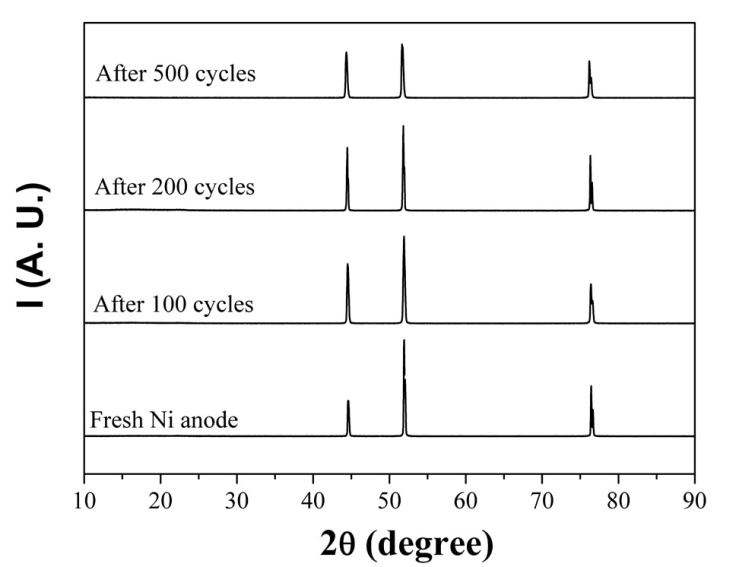
**

**Figure S9** XRD patterns of nickel film anode before and after cycles

**Part 4 Nickel ion battery**

**The preparation of nickel ion battery**

The prototype nickel ion battery with α-MnO_2_ cathode, 1 mol L^-1^ NiSO_4_ aqueous electrolyte, a fiber paper separator and a nickel foam anode as shown in Figure S10. The nickel foam is used due to its larger surface area than plate.


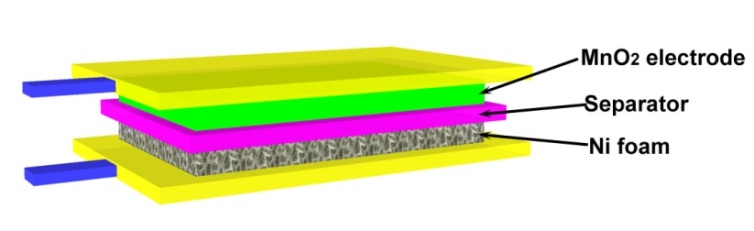


**Figure S10** Cell structure of nickel ion battery

Electrochemical tests were performed with Solartron 1480 electrochemical station and Land CT2001A equipment. The discharge capacity of the cell is calculated according to the formula:

*C* = *I t / m* (S-8)

Where *C* is specific capacity (milliampere·hour per gram, mA·h g^-1^), *I* is the applied current (milliampere, mA), *t* is discharge time (hour, h), and *m* is the mass of the active material (gram). The energy density is calculated by the following equation:

*E* = C V (S-9)

Where *C* is specific capacity (mA·h g^-1^) and V is the average voltage of battery. The average voltage of NIB and ZIN is 0.85 and 1.45 V.

The energy density of zinc ion battery (ZIB) and nickel ion battery (NIB) are listed in Table S2.

Table S2 Theoretical energy density of ZIB and NIB

|  | Measured positive MnO_2_ capacity (mAh/g) | Negative capacity  (mAh/g) | Average voltage  (V) | Energy density of entire battery  (Wh/kg) |
| --- | --- | --- | --- | --- |
| NIB | 298 | 913 | 0.85 | 342 |
| ZIB | 220 | 720 | 1.45 | 320 |

**Electrochemical performance**

The prototypes of nickel ion battery (NIB) have been assembled according to the structure as shown in the insert of Figure S11. The charge/discharge curves of NIB at a current density of 0.1 ampere per gram indicates a capacity of ca. 160 milliampere hours per gram (mAh g^-1^) .


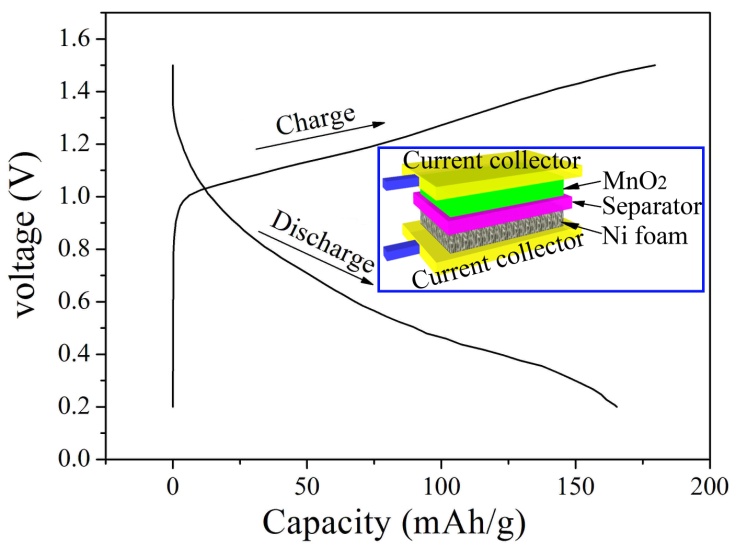


**Figure S11** Charge/discharge curve of nickel ion battery at a current density of 0.1 Ampere per gram. The insert shows the battery structure of nickel ion battery.

The continuous rechargeability of NIB has been investigated by the galvanostatic charge/discharge cycling tests at 100% depth of discharge. Figure S12 shows the raw data of continuous charge/discharge curves.


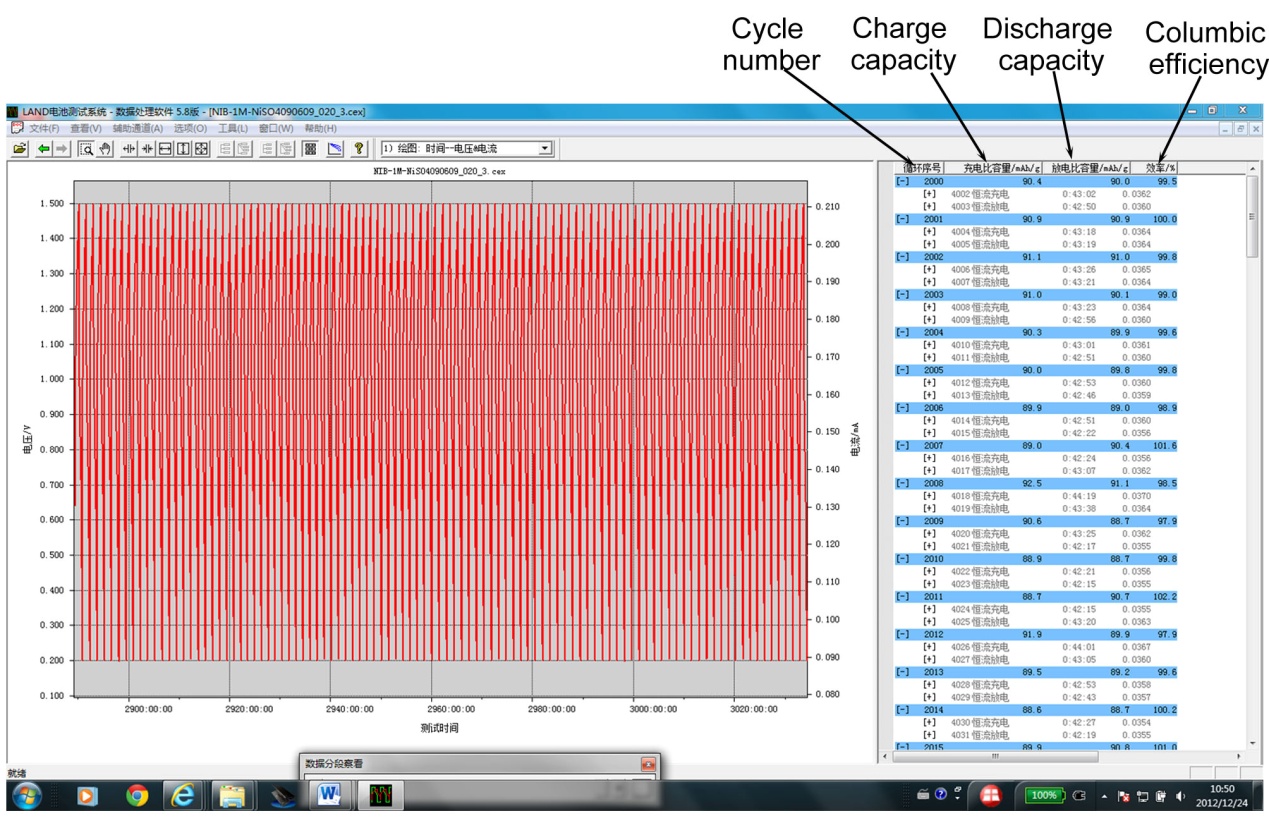


**Figure S12** Raw data from Land (Chinese version) for cycle life. Left shows the continuous charge/discharge curves from 2000th cycle to 2100th cycle. Right exhibits the charge capacity, discharge capacity and coulombic efficiency.

The Columbic efficiency of NIB for 2200 cycles is shown in Figure S13. NIB shows a good Columbic efficiency.


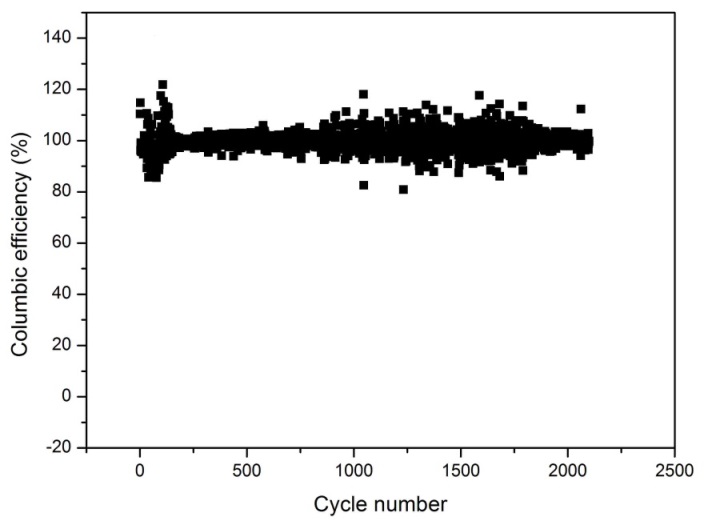


**Figure S13** Columbic efficiency of NIB for 2200 cycles

**Part 5 Zinc ion battery**

The concept and battery chemistry of zinc ion battery (ZIB) can be seen in our published papers (*Angew. Chem. Int. Ed. 2012, 51: 933-935*). It is obvious that the battery chemistry of rechargeable zinc ion battery is totally different from that of primary Zn/MnO_2_ battery so as to obtain totally different electrochemical performance. Primary Zn/MnO_2_ batteries is capable of discharge at very low rates, while secondary ZIB is capable of charge or discharge at very high rates.

Figure S14 shows the cyclic voltammogram (CV) plot of ZIB. There are only two anodic and cathodic peaks emerged around 1.3 and 1.7 V, respectively. Cyclic voltammogram of integral cell indicated that the insertion and extraction potentials of zinc ion occur in accordance with the single α-MnO_2_ electrode measurements.

**
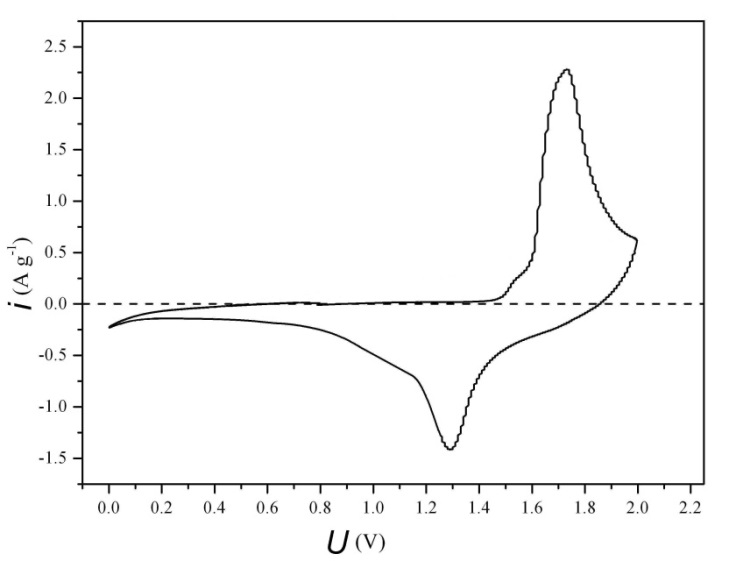
**

**Figure S14** CV plot of ZIB

The continuous rechargeability of ZIB has been investigated by the galvanostatic charge/discharge cycling tests at 100% depth of discharge. Figure S15 shows the continuous charge/discharge curves of ZIB at a power density of 1450 W Kg^-1^. The first discharge capacity of ZIB is 187 mAh g^-1^. The energy density is 270 Wh Kg^-1^. It is shown that ZIB can be continuously charged and discharged even at a relatively high power. The rate ability of ZIB has also been investigated by the galvanostatic charge-discharge cycling tests. Figure S15a shows the charge/discharge cycling curves at current densities 0.1 A g^-1^.

**
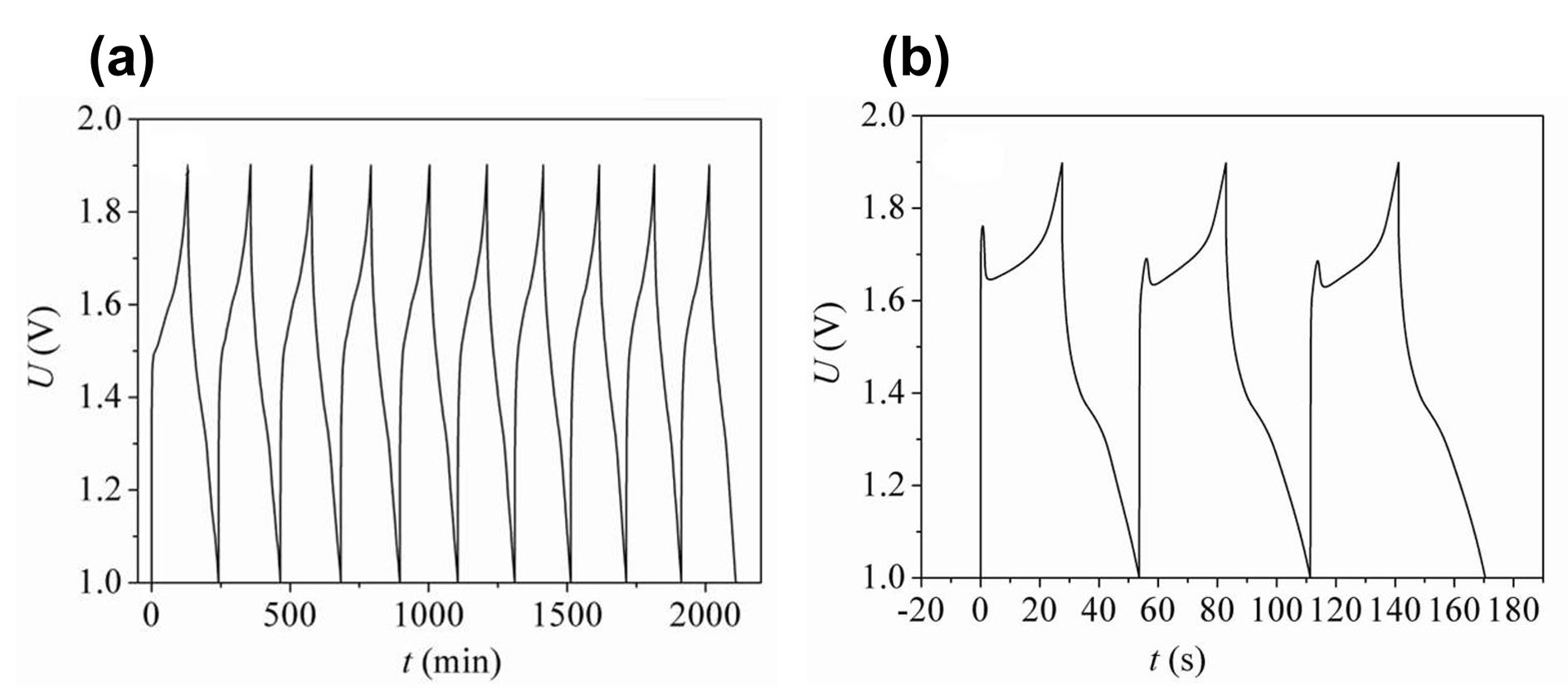
**

**Figure S15** (a) Charge/discharge curves of ZIB at low current density. (b) The charge/discharge curves of ZIB within 30 seconds.

Figure S15b shows the charge/discharge cycling curves at current densities 8.0 A g^-1^. It is shown that the ZIB can still be charged or discharged within 27 seconds. It is well known that none of the current batteries is able to be continuously charged or discharged within tens of seconds. The fast charge ability of ZIB has solved the bothering questions of extremely slow charge of batteries for over centuries and will ultimately make human lives more convenient.

**Part 6 MnO_2_/activated carbon cells**

The detailed information of MnO_2_/activated carbon (AC) cells can be seen in the papers published by us and other researchers. The MnO_2_/AC cells used the coin cell assembly consisting of MnO_2_ positive and AC negative electrodes. The MnO_2_/AC weight ratios were calculated by considering specific voltammetric charge and the appropriate potential window of positive and negative electrodes in different electrolytes. A glass paper was used as the separator. The stainless steel foil (30 μm in thickness) was used as the current collector. All the electrolytes were bubbled by N_2_ gas for 30 min to eliminate oxygen before assembly. The weight ratio between positive MnO_2_ and negative AC is shown in the Table S3 for each Mg^2+^, Ca^2+^, Ba^2+^, or La^3+^ ion.

**Table S3** Weight ratio between positive MnO_2_ and negative AC

| **Cation species** | **Positive MnO_2_ electrode** | | **Negative AC electrode** | | **Mass ratio** |
| --- | --- | --- | --- | --- | --- |
|  | **SC**  **(F g^-1^)** | **Potential window (V)** | **SC (F g^-1^)** | **Potential window (V)** |  |
| **Mg** | **325** | **0.9** | **72** | **1.1** | **3.69** |
| **Ca** | **314** | **0.9** | **80** | **1.1** | **3.17** |
| **Ba** | **281** | **0.9** | **76** | **1.1** | **3.03** |
| **La** | **295** | **0.9** | **71** | **1.1** | **3.40** |

The structure of MnO_2_/AC cells is shown in Figure S16. The structure of MnO_2_/AC cells is similar to that of NIB or ZIB.

MnO_2_ electrode

Current collector

Separator

AC electrode

Current collector

**Figure S16** Structure of MnO_2_/AC cells

Figure S17a shows the charge/discharge curves of MnO_2_/AC cells with Mg^2+^, Ca^2+^, or Ba^2+^ as energy storage in cathode, respectively. Figure S17b shows the cycle life of MnO_2_/AC cells with Mg^2+^, Ca^2+^, or Ba^2+^ as energy storage in cathode, respectively. The MnO_2_/AC cells all shows the good cycle life over 2000 times.

**
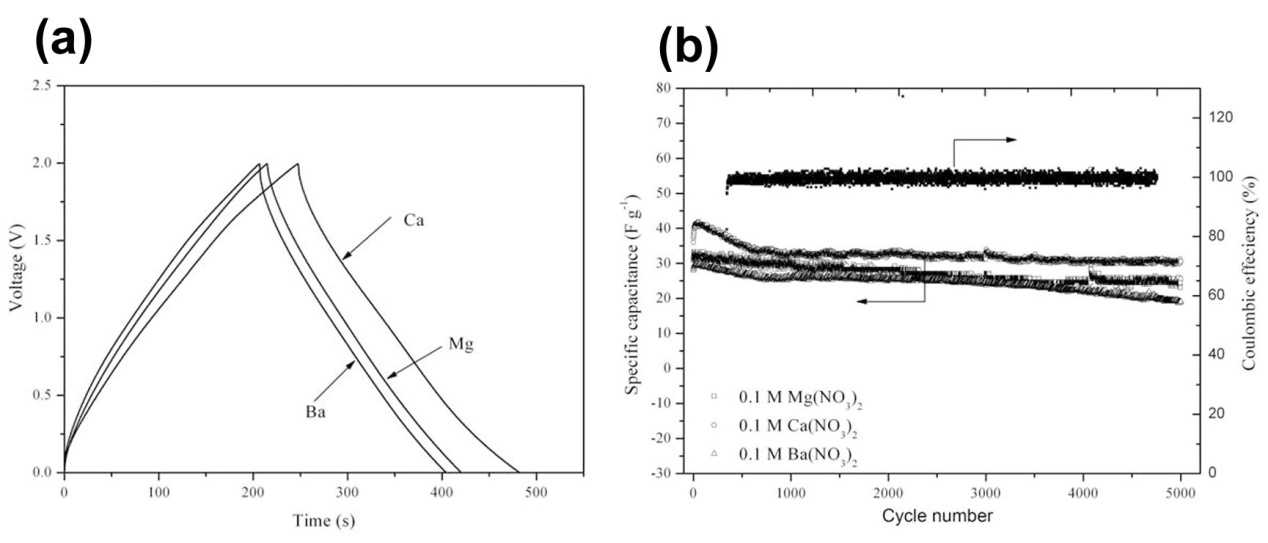
**

**Figure S17** Charge/discharge curves (a) and cycle life (b) of MnO_2_/AC cells with Mg^2+^, Ca^2+^, or Ba^2+^ as energy storage in cathode respectively.

**Part 7 Diffusion coefficient**

The diffusion coefficient of each ion is determined by electrochemical impedance spectrum. The electrochemical impedance spectroscopy (EIS) was conducted at 1.0 V vs. SCE by sweeping frequencies from 10 mHz to 10 KHz. The measured impendence data were analyzed by using Zview software. The diffusion coefficient was calculated based on the radius ～ 2 nm of MnO_2_ particle and the expression of the Warburg impendence.


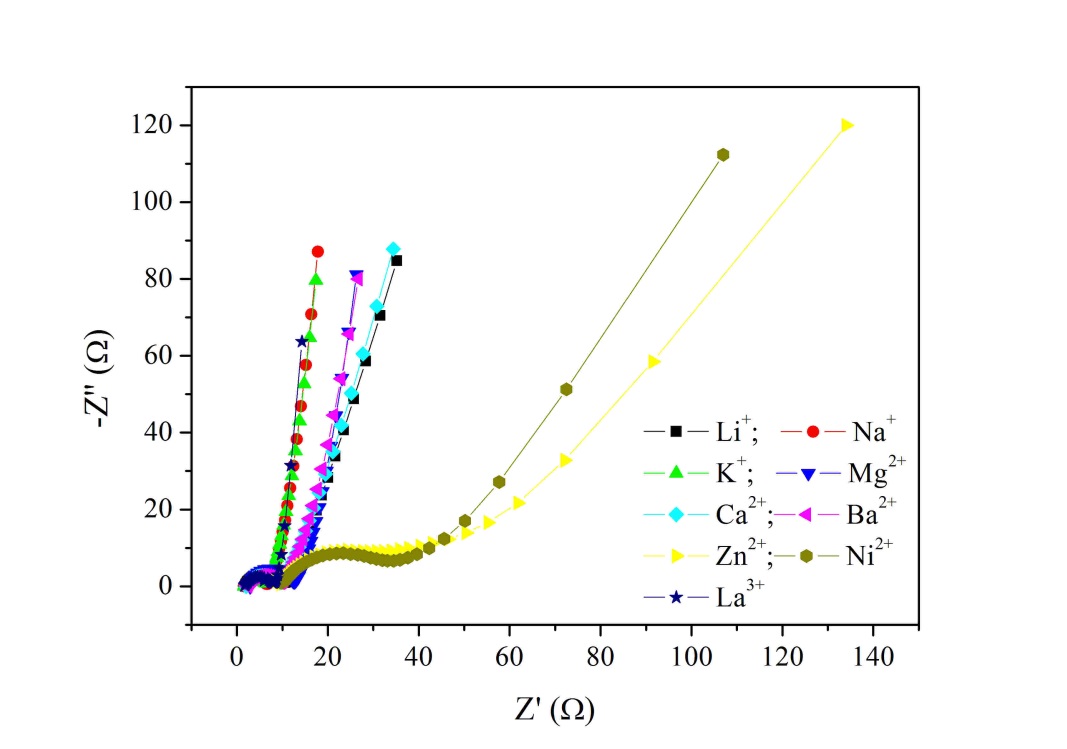
**Figure S18** Nyquist plot for each ion

The Nyquist plot of each ion is shown in Figure S18. The model circuit consists of four elements: the internal resistance (*R*_s_), the constant phase element (CPE) used in place of double layer capacitance (*C*_dl_), the charge transfer resistance (*R*_ct_), and the Warburg impendence (*Z*_w_). The internal resistance (*R*_s_) includes the bulk electrolyte solution resistance, the intrinsic resistance of active material, and the electron transfer resistance at current collector/electrode boundary. The constant phase element (CPE) is used in place of double layer capacitance (*C*_dl_) at the electrode-electrolyte boundary because the non-ideal natures of capacitance arise due to inhomogeneous nature of electrode. The charge transfer resistance (*R*_ct_) represents the kinetic resistance to charge transfer at electrode-electrolyte boundary or intrinsic charge transfer resistance of porous electrode. The Warburg impendence (*Z*_w_) associates with the diffusion of cation in the bulk electrode.

Detailed information could be seen in the reference (*Journal of Power Sources, 2011, 196: 7854-7859*).


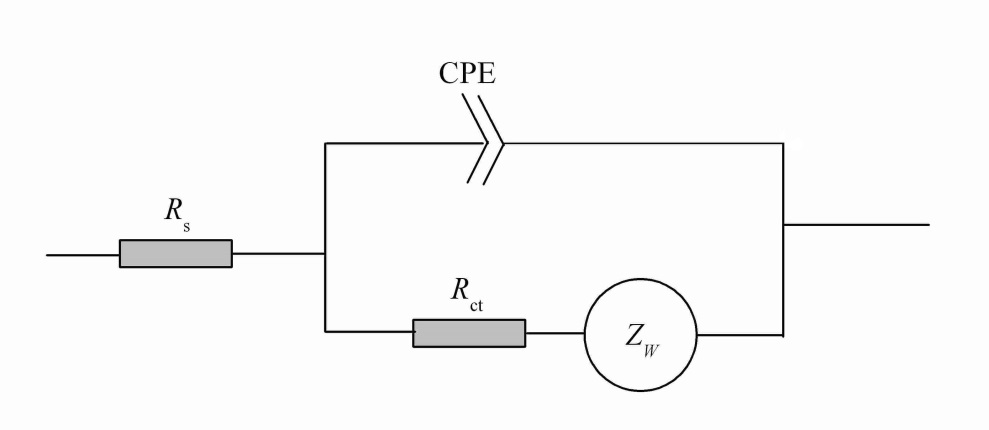
**Figure S19** Equivalent series circuit
